# Development and Validation of a Prediction Model for Irreversible Worsened Cardiac Function in Patients With Acute Decompensated Heart Failure

**DOI:** 10.3389/fcvm.2021.785587

**Published:** 2021-12-10

**Authors:** Lei Wang, Yun-Tao Zhao

**Affiliations:** Department of Cardiology, Aerospace Center Hospital, Beijing, China

**Keywords:** acute decompensated heart failure, irreversible worsened cardiac function, prediction model, development and validation, nomogram

## Abstract

**Background:** Irreversible worsening of cardiac function is an adverse event associated with significant morbidity among patients with acute decompensated heart failure (ADHF). We aimed to develop a parsimonious model which is simple to use in clinical settings for the prediction of the risk of irreversible worsening of cardiac function.

**Methods:** A total of 871 ADHF patients were enrolled in this study. Data for each patient were collected from the medical records. Irreversible worsening of cardiac function included cardiac death within 30-days of patient hospitalization, implantation of a left ventricular assistance device, or emergency heart transplantation. We performed LASSO regression for variable selection to derive a multivariable logistic regression model. Five candidate predictors were selected to derive the final prediction model. The prediction model was verified using C-statistics, calibration curve, and decision curve.

**Results:** Irreversible worsening of cardiac function occurred in 7.8% of the patients. Advanced age, NYHA class, high blood urea nitrogen, hypoalbuminemia, and vasopressor use were its strongest predictors. The prediction model showed good discrimination C-statistic value, 0.866 (95% CI, 0.817–0.907), which indicated good identical calibration and clinical efficacy.

**Conclusion:** In this study, we developed a prediction model and nomogram to estimate the risk of irreversible worsening of cardiac function among ADHF patients. The findings may provide a reference for clinical physicians for detection of irreversible worsening of cardiac function and enable its prompt management.

## Background

Approximately 4 million people in China were estimated to have heart failure, with a prevalence of 0.4–1.3% (1). Patients hospitalized with acute decompensated heart failure (ADHF) have a high risk of mortality, with 30-day mortality rates approaching 10% (2). Some patients are discharged from the hospital after treatment, while in others deterioration of heart failure occurs during hospitalization, and remaining patients show irreversible worsening of heart function. Patients who experienced in-hospital worsening heart failure had high 30-day mortality (29.7%) (3). Therefore, it is necessary to predict the clinical course of heart failure as early as possible, to enable the selection of evidence-based management strategies to improve the treatment and nursing of patients with heart failure.

Risk prediction models are often used to classify patients and simplify treatment decisions. They help physicians predict prognoses and interpret the results of prognostic studies to improve the level of care for inpatients with heart failure.

To date, many death prediction models for ADHF have been developed and verified (4–7). However, the ability of these models to predict individual patient outcomes is limited. Many of these models only show moderate effects (mortality based on C-statistics: 0.70–0.80) (4, 8). Calibration is also poor even after global recalibrations (9, 10). So their utility is limited in clinical settings.

We, therefore, aimed to develop a practical risk prediction model for irreversible worsening of cardiac function among ADHF patients.

## Methods

The Transparent Reporting for Individual Prognosis or Diagnosis (TRIPOD) recommendation was used as the guideline for the development and validation of the multivariable prediction model (11).

### Data Sources and Processing

This study was approved by the ethics committee of the Aerospace Center Hospital, Beijing, China. Written informed consent was waived owing to the use of anonymous retrospective data. Demographic, clinical, and laboratory data were retrieved from the electronic hospital database. A team of experienced clinical cardiologists reviewed and cross-checked the data. Each record was independently verified by two clinicians.

### Patient Selection

A total of 1,222 patients diagnosed with ADHF who were admitted to the Aerospace Center Hospital (a tertiary hospital in Beijing, China) between January 2017 to December 2020 were recruited retrospectively. ADHF was diagnosed following the guidelines of the European Society of Cardiology (12).

**Figure 1:**
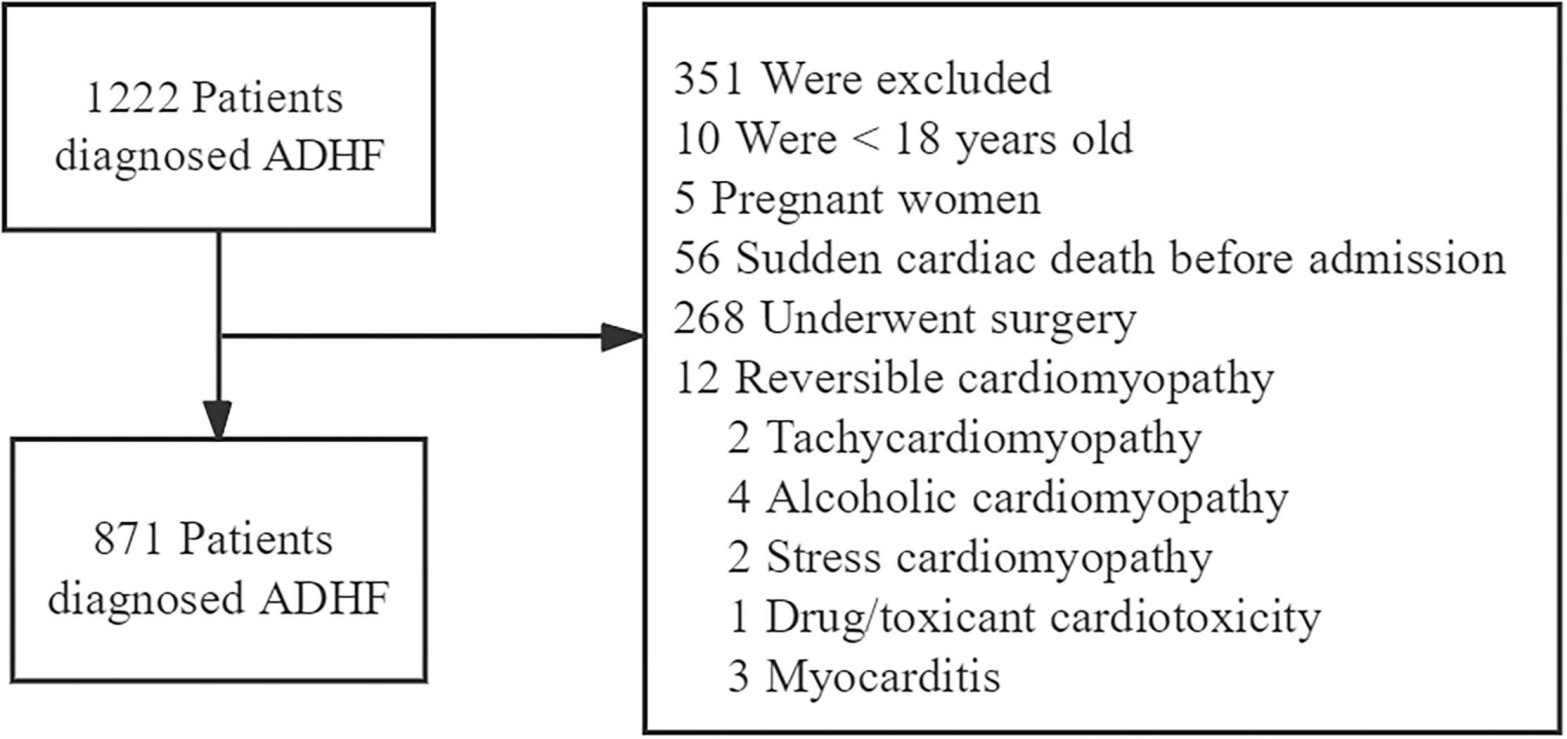
Flow chart demonstrating the process of selection from a total of 1,222 patients.

We excluded patients according to the following criteria: <18 years old, pregnant women, sudden cardiac death before admission, those who underwent surgery, reversible cardiomyopathy (tachycardiomyopathy, alcoholic cardiomyopathy, stress cardiomyopathy, drug/toxicant cardiotoxicity, and myocarditis) (Figure 1).

### Potential Predictive Variables

Consistent data for each patient were collected from the medical records, and all candidate predictors were selected based on a detailed literature review and clinical evidence within the confines of data availability.

Demographic variables included sex, age, height, and weight values. Medical history included the presence of diabetes, hypertension, coronary artery disease, previous heart failure, atrial fibrillation, previous renal dysfunction, cerebral infarction, cancer, and cirrhosis. Clinical signs and symptoms included categorical and continuous variables as follows: New York Heart Association (NYHA) functional class, paroxysmal nocturnal dyspnea, orthopnea, heart rate, systolic blood pressure, diastolic blood pressure, rales (>1/2 lung fields), jugular venous distension, peripheral edema. Imaging results consisted of left ventricular ejection fraction (LVEF) by two-dimensional transthoracic echocardiography, and laboratory findings included evaluation of levels of B-type natriuretic peptide, troponin I, hemoglobin, C-reactive protein, alanine aminotransferase, blood urea nitrogen, creatinine, albumin, serum sodium, serum potassium, uric acid, and glucose. We recorded the baseline values of these tests, with the first value taken within 2 days of onset admission. The treatment regime included administration of aldosterone antagonists, loop diuretic, angiotensin-converting enzyme inhibitors/angiotensin receptor blockers (ACE-Is/ARBs), beta-blockers, anticoagulants, aspirin, ADP-P2Y12 antagonists, and/or vasopressors. Detailed and specific definitions of the variables included are listed in Supplementary Table 1.

### Characteristics of Irreversible Worsening of Cardiac Function

Irreversible worsening of cardiac function included cardiac death, implantation of a left ventricular assistance device, or emergency heart transplantation within 30-days of hospitalization.

### Sample Size

We considered the events per variables (EPV) ratio between 5 and 10 acceptable, with EPV of 10 as the optimal number to minimize the overfitting of the regression model (13). According to this rule, we required a total of 50 ADHF inpatients who exhibited irreversible worsening of cardiac function to evaluate five candidate predictors. Assuming that the prevalence of 30-day mortality was ~10% among patients with ADHF (14, 15), a total sample size of at least 500 would suffice. Thus, to ensure an adequate number of events, we decided to collect data of at least 500 individuals.

### Handling Missing Data

Before data analysis, predictor variables were inspected for missing values. Among the predictors, the proportion of missing data was 0.34–6.2%. To include these data in the analyses, we imputed missing data through multiple imputations using chained equations of the mice package in R, in which predictive mean matching is embedded with the cases (k) = 5 as the default. Baseline clinical characteristics before and after imputations are listed in Supplementary Table 2.

### Statistical Analysis

Data were presented as frequencies (percentages) for categorical variables and as mean (standard deviation) or median (interquartile ranges [IQRs]) for continuous variables. Means for continuous variables were compared using *t*-tests when the data were normally distributed; else, the Mann-Whitney U test was used. Proportions of categorical variables were compared using the χ2 test; the Fisher exact probability test was used when the data were limited. The statistical significance level for all tests was set as α = 0.05; *P* <0.05 (two-tailed) was considered statistically significant.

### Variable Selection

The least absolute shrinkage and selection operator (LASSO) regression is a compression estimation used for collinearity estimates between covariates. When there are several collinear predictors, LASSO selects only one and ignores the others or zeroes out some regression coefficients. The glmnet package in R was used for LASSO regression analysis, the lambda values were selected after 10-fold-cross-validation; the larger the lambda value, the more compact was the model. Briefly, for cross-verification, the data were divided into 10 equal parts. First, the whole data were fit to generate a lambda sequence. Second, one data point was excluded each time, and the remaining nine were used for verification. The averages and standard deviations of the deviance obtained after 10 time-verification were calculated. Finally, two models were obtained as the output. One was based on lambda.min, that is, the lambda whose deviance mean was the smallest; the other was based on lambda.1se, that is, the maximum lambda corresponding to the deviance mean within one standard deviation of the minimum value.

The results of the analysis were considered in conjunction with clinical evidence, sample size, and statistical ability (16). Finally, five candidate predictors were selected to derive the prediction model and build a nomogram based on the results of the logistic regression model.

### Model Validation

We performed internal validation of the model development processes using the bootstrap resampling method (500 bootstrap samples per model) to obtain an unbiased estimate of model performance (16). Then the prediction model was verified using C-statistics, calibration curve (17), and decision curve analysis (18).

### Sensitivity Analysis

Given the heterogeneity in sex (19, 20) and history of coronary artery disease (21), to investigate whether the predictive strength of the nomogram changed due to these predictors, we evaluated the C-statistics for the subgroups.

### Statistical Analysis Software

Data were analyzed using statistical packages in R (The R Foundation; http://www.r-project.org; version 4.0.5).

## Results

In total, 871 ADHF patients were included in this study; the mean age was 75.30 years, and 412 (47.30%) patients were male. Sixty eight patients had irreversibly worsened cardiac function, which implied that the incidence was 7.80%.

Unlike other patients, those with irreversible worsening of cardiac function were older (*P* <0.05). Table 1 shows the comparison of the patient characteristics in the study.

**Table 1:** Comparison of the characteristics between the reversible worsen and irreversible worsen groups.

**Variables**	**Overall**	**Reversible worsen**	**Irreversible worsen**	***P*-value**
*N*	871	803 (92.19%)	68 (7.81%)	
Sex, Male	412 (47.30%)	378 (47.07%)	34 (50.00%)	0.643
Age, years	75.30 (12.54)	74.96 (12.75)	79.29 (8.97)	0.005
BMI (kg/m^2^)	24.34 (4.31)	24.39 (4.36)	23.73 (3.64)	0.222
Diabetes	370 (42.48%)	331 (41.22%)	39 (57.35%)	0.010
Hypertension	605 (69.46%)	562 (69.99%)	43 (63.24%)	0.246
Coronary artery disease	587 (67.39%)	533 (66.38%)	54 (79.41%)	0.028
Previous congestive heart failure	283 (32.49%)	254 (31.63%)	29 (42.65%)	0.063
Atrial fibrillation	356 (40.87%)	330 (41.10%)	26 (38.24%)	0.645
Previous renal dysfunction	158 (18.14%)	138 (17.19%)	20 (29.41%)	0.012
Cerebral infarction	178 (20.44%)	167 (20.80%)	11 (16.18%)	0.364
Cancer	96 (11.02%)	84 (10.46%)	12 (17.65%)	0.069
Cirrhosis	11 (1.26%)	10 (1.25%)	1 (1.47%)	0.873
NYHA classification				<0.001
2	242 (27.78%)	236 (29.39%)	6 (8.82%)	
3	401 (46.04%)	376 (46.82%)	25 (36.76%)	
4	228 (26.18%)	191 (23.79%)	37 (54.41%)	
Paroxysmal nocturnal dyspnea	166 (19.06%)	151 (18.80%)	15 (22.06%)	0.512
Orthopnoea	168 (19.29%)	140 (17.43%)	28 (41.18%)	<0.001
Heart rate (beats/min)	89.60 (22.60)	88.81 (22.23)	98.84 (24.91)	<0.001
Systolic blood pressure (mmHg)	130.84 (24.60)	132.08 (24.33)	116.21 (23.16)	<0.001
Diastolic blood pressure (mmHg)	72.65 (16.68)	73.42 (16.73)	63.63 (13.15)	<0.001
Rales (>1/2 lung fields)	361 (41.45%)	314 (39.10%)	47 (69.12%)	<0.001
Jugular venous distension	168 (19.29%)	143 (17.81%)	25 (36.76%)	<0.001
Peripheral edema	559 (64.18%)	503 (62.64%)	56 (82.35%)	0.001
LVEF (%)	51.51 (11.79)	51.70 (11.80)	49.32 (11.60)	0.111
B-type natriuretic peptide (pg/ml)	786.00 (331.50–1601.00)	743.00 (321.00–1535.00)	1442.50 (656.50–2458.75)	<0.001
Troponin I(ng/ml)	0.05 (0.04–0.10)	0.05 (0.04–0.10)	0.10 (0.05–0.66)	0.004
Hemoglobin (g/L)	116.13 (24.62)	117.27 (24.27)	102.72 (24.93)	<0.001
C-reactive protein (mg/L)	10.00 (4.00- 27.14)	9.00 (3.74–25.29)	21.94 (11.98–65.57)	<0.001
Alanine aminotransferase (IU/L)	16.40 (11.20–27.85)	16.10 (11.20–27.70)	19.10 (10.90–30.38)	0.250
Blood urea nitrogen (mmol/L)	8.10 (5.90–11.70)	7.90 (5.80–11.20)	12.86 (8.38–19.75)	<0.001
Creatinine (μmol/L)	92.40 (72.90–127.45)	90.00 (72.50–122.25)	133.15 (84.27–172.30)	0.003
Albumin (g/L)	37.33 (105.51)	35.83 (4.75)	32.00 (5.41)	<0.001
Sodium (mmol/L)	138.43 (5.60)	138.42 (5.05)	138.59 (10.06)	0.806
Potassium (mmol/L)	4.23 (0.62)	4.21 (0.61)	4.45 (0.70)	0.002
Uric acid (μmol/L)	392.80 (296.65–514.20)	393.60 (297.00–510.85)	374.30 (289.75–546.25)	0.572
Glucose (mmol/L)	8.40 (4.21)	8.34 (4.13)	9.18 (5.09)	0.112
Aldosterone antagonists	643 (73.82%)	599 (74.60%)	44 (64.71%)	0.075
Loop diuretic	804 (92.31%)	744 (92.65%)	60 (88.24%)	0.189
ACE-Is/ARBs	332 (38.12%)	317 (39.48%)	15 (22.06%)	0.005
Beta-blockers	602 (69.12%)	569 (70.86%)	33 (48.53%)	<0.001
Anticoagulants	259 (29.74%)	248 (30.88%)	11 (16.18%)	0.011
Aspirin	376 (43.17%)	345 (42.96%)	31 (45.59%)	0.675
ADP-P2Y12 antagonists	370 (42.48%)	336 (41.84%)	34 (50.00%)	0.191
Vasopressor	86 (9.87%)	50 (6.23%)	36 (52.94%)	<0.001

*BMI, body mass index; NYHA, New York Heart Association; LVEF, left ventricular ejection fraction; ACE-Is/ARBs, angiotensin converting enzyme inhibitors/Angiotensin Receptor Blockers; ADP, adenosine diphosphate*.

### Variable Selection and Model Development

Based on the LASSO analysis (Supplementary Figure 1), we identified model 1 consisting of four variables. The prediction model can accommodate five variables. Since age was an important factor affecting the prognosis of patients (6, 22), it was included in the model (16). Then we derived the prediction model 2 with the five variables (Table 2). We evaluated the C-statistics for each model (Table 3).

**Table 2:** Variables and regression coefficients of the two models.

**Model**	**1**	**2**
Approach	LASSO	LASSO + Age
(Intercept)	0.28111	−2.58620
Age (years)		0.03388
NYHA class 3	0.92603	0.97600
NYHA class 4	0.10359	0.12549
Blood urea nitrogen (mmol/L)	0.06791	0.06832
Albumin (g/L)	−0.12590	−0.11930
Vasopressor	2.53253	2.56681

*LASSO, least absolute shrinkage and selection operator; NYHA, New York Heart Association*.

**Table 3:** Comparison of the two models.

**Model**	**1**	**2**
Approach	LASSO	LASSO + Age
C-Statistics	0.859	0.866
95% CI low	0.803	0.817
95% CI up	0.903	0.907
Best threshold	−3.009	−2.696
Specificity	0.722	0.784
Sensitivity	0.838	0.779

*LASSO, least absolute shrinkage and selection operator*.

**Figure 2:**
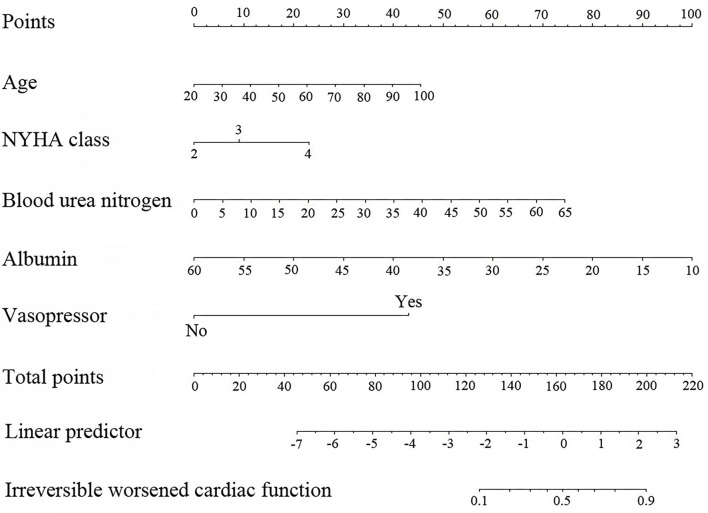
Nomogram based on the results of for logistic regression model (age in years, blood urea nitrogen in g/L, and albumin in g/L).

Finally, we selected model 2 with five candidate predictors to derive the prediction model and built a nomogram based on the logistic regression model (Figure 2).

The risk score was calculated for each patient using the following formula derived from the expression of the five variables weighed by their regression coefficients: Risk score = −4.29800 +0.03388 × Age (years) +0.53645 × NYHA class +0.30738 × NYHA class = 4 +0.06832 × Blood urea nitrogen (g/L) −0.11930 × Albumin (g/L) +2.56681 × (Vasopressor = 1).

### Model Validation

**Figure 3:**
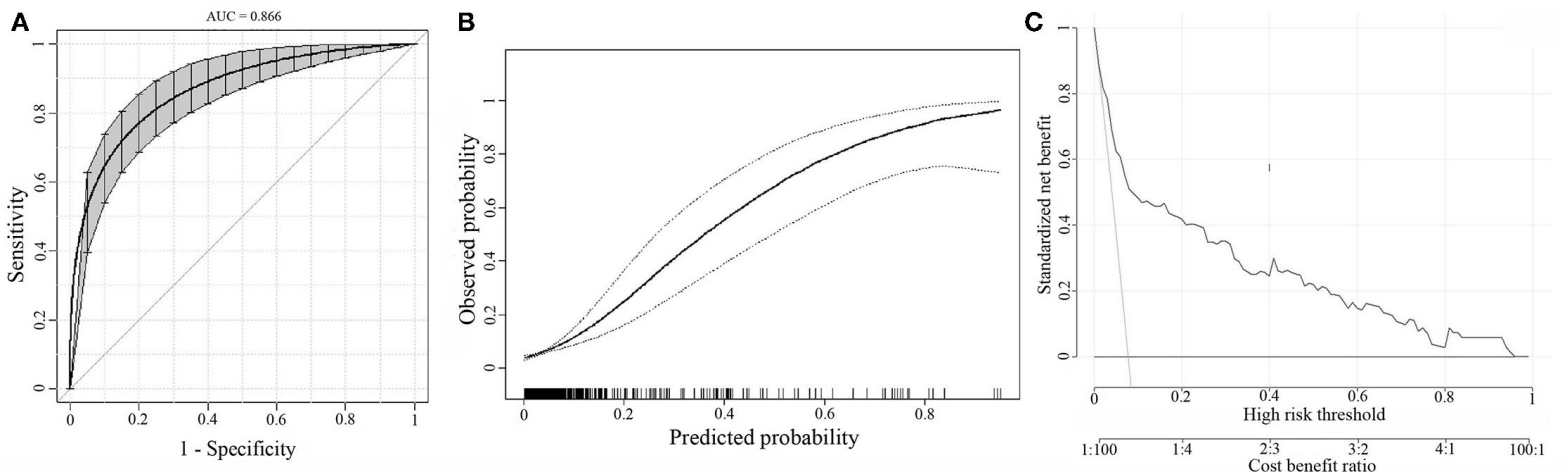
Internal model validation. **(A)** The discrimination of the prediction model by the bootstrap method. **(B)** Calibration plots of the model by the bootstrap method. **(C)** Decision curve analysis for the model.

The bootstrap analysis showed a good discriminative ability for the prediction model (C-statistics: 0.866 [95% CI, 0.817–0.907]) (Figure 3A). The calibration plots of the model based on the bootstrap method showed good performance (Figure 3B). Decision curve analysis showed moderate clinical efficacy of the model (Figure 3C).

### Sensitivity Analysis

The discrimination of the prediction model was consistent for the sex and coronary artery disease history subgroups (C-statistics for the male subgroup: 0.827 [95% CI, 0.748–0.906], C-statistics for the female subgroup: 0.912 [95% CI, 0.868–0.956], C-statistics for the coronary artery disease subgroup: 0.859 [95% CI, 0.802–0.916], C-statistics for without coronary artery disease subgroup: 0.907 [95% CI, 0.846–0.967]) (Supplementary Table 3).

### Nomogram Interpretation

The point in Figure 2 was the selected scoring standard or scale. For each independent variable, a straight line perpendicular to the point's axis (through a ruler) was made. The intersection point represented the score for the value of the independent variable. For example, age at 60 meant 22.5 points and NYHA class at (IV) meant 22.5 points. The corresponding points of these independent variables for each patient were calculated and the total points were estimated; thus the perpendicular line location to the axis was estimated. This indicated the risk of irreversible worsening of cardiac function in the corresponding patient.

## Discussion

Based on the LASSO regression, we found four predictive risk factors for irreversible worsening of cardiac function, including NYHA class, high blood urea nitrogen, hypoalbuminemia, and use of vasopressor, in this retrospective study. Since age was an important factor affecting the prognoses of patients, this variable was also added (23).

We took many steps to minimize the potential bias (24). This model applies to a broad spectrum of patients with heart failure, including those with preserved left ventricular systolic function, the ones diagnosed with ADHF, or newly diagnosed with acute heart failure, and those previously diagnosed with heart failure.

The discussion on variable screening has been an ongoing one. The analysis results, clinical reasons, sample size, and statistical power are simultaneously considered. The predictive variables in the nomogram model are convenient for clinical acquisition and thus, the construction of these models is feasible. We performed LASSO regression analysis to select variables. This parsimonious model showed sufficiently stable applicability. Sometimes machine learning algorithms are used to construct models and most of them are non-parametric. However, owing to the absence of parameters like regression coefficients, the clinical interpretation of such non-parametric models is difficult (23). Therefore, we did not use machine learning algorithms for variable filtering.

Interestingly, although B-type natriuretic peptide is a widely recognized prognostic factor for patients with heart failure (25), it has not been included in the risk prediction models. The B-type natriuretic peptide is affected by several factors; for example, B-type natriuretic peptide levels are lower in obese people (25), thus, it is only available in some of the enrolled patients. Although B-type natriuretic peptide has an accurate prognostic ability in inpatients with heart failure, other clinical factors may also play a key role in influencing prognosis. Further analysis showed that B-type natriuretic peptide does not contribute significantly to the nomogram to indicate the poor score. Therefore, considering the major risk factors in the risk scoring system is a necessary condition for predicting important outcomes (23).

Consistent with previous studies, the renal function also was an important predictor of outcomes (26). Serum urea nitrogen level was a stronger predictor than creatinine level. The greater prognostic power of serum urea nitrogen level may be attributed to its incorporation in both prerenal and renal function statuses (27). Serum urea nitrogen level is a factor incorporated in other predictive models, such as blood urea nitrogen level for predicting in-hospital mortality (6, 22) and post-discharge clinical outcomes (4). Heart and kidney interactions are complex, and the subject is of immense clinical and scientific interest and debate. The coexistence of acute cardiac and renal dysfunction, termed acute cardiorenal syndrome, is correlated with increased mortality and results in adverse outcomes (27).

### Limitations

In addition to these findings, some limitations exist in this study which should be addressed in the future. First, this was an observational study, and thus, we could not draw direct causal conclusions. Second, we excluded the patients with reversible cardiomyopathy and those who underwent surgery. Hence, the findings of this study cannot be extrapolated to the general population. Third, the non-linearity and interaction of variables were not analyzed. This may need to be investigated in the future. Finally, as this study was based on patients from a single center in China, there existed an inevitable sample selection bias, and external validation of the findings was lacking. As such, prospective validation to examine model stability, reproducibility, and external verification in independent samples is needed.

We evaluated ADHF cardiac function in patients using the irreversible worsening prediction model. This would allow for early prediction of the patient's clinical course, and allocation of appropriate resources, including transplantation and mechanical circulation auxiliary equipment.

## Conclusion

In conclusion, an irreversible worsening of cardiac function is an adverse event associated with significant morbidity among patients with ADHF. Currently, there is no effective practical tool for estimating its occurrence likelihood. In this study, we developed a risk score-based prediction model and nomogram to estimate the risk of irreversible worsening of cardiac function among ADHF patients. The findings may provide a reference for clinical physicians to detect irreversible worsening of cardiac function and manage it promptly.

## Data Availability Statement

The original contributions presented in the study are included in the article/Supplementary Material, further inquiries can be directed to the corresponding author/s.

## Ethics Statement

The studies involving human participants were reviewed and approved by the Ethics Committee of the Aerospace Center Hospital, Beijing, China. Written informed consent for participation was not required for this study in accordance with the national legislation and the institutional requirements.

## Author Contributions

Y-TZ and LW made contributions to data collection, drafted the manuscript, and made contributions to the analysis and interpretation of the data. All authors contributed to the article and approved the submitted version.

## Conflict of Interest

The authors declare that the research was conducted in the absence of any commercial or financial relationships that could be construed as a potential conflict of interest.

## Publisher's Note

All claims expressed in this article are solely those of the authors and do not necessarily represent those of their affiliated organizations, or those of the publisher, the editors and the reviewers. Any product that may be evaluated in this article, or claim that may be made by its manufacturer, is not guaranteed or endorsed by the publisher.
