# Supplementary material for: Development and Validation of a Prediction Model for Irreversible Worsened Cardiac Function in Patients With Acute Decompensated Heart Failure
Source: Front Cardiovasc Med. 2021 Dec 10;8:785587. doi: 10.3389/fcvm.2021.785587 (PMC8702716; doi:10.3389/fcvm.2021.785587)
Supplement: Supplementary file 1 [file Data_Sheet_1.pdf]

Supplemental Material

Table S1. Definition of variables.

Variables	Method of assessment and grading	Reference
**Demographic information**		
Sex	Female<br>Male	Female vs male
Age	Assessed at admission	In units of years
Height	Assessed at admission	In units of m
Weight	Assessed at admission	In units of Kg
**Medical History**		
Diabetes	Diabetes mellitus, Type 1 or 2 diabetes, taking hypoglycemic medications	Absence vs presence
Hypertension	Hypertension, primary or secondary Hypertension, hypertension grade 1, 2 or 3	Absence vs presence
Coronary artery disease	Coronary artery disease, angina, myocardial infarction, ischemic heart disease, coronary artery stent implantation	Absence vs presence
Previous heart failure	Heart failure, systolic heart failure, diastolic heart failure, cardiac dysfunction, heart failure unspecified	Absence vs presence
Atrial fibrillation	Atrial fibrillation, paroxysmal or persistent atrial fibrillation	Absence vs presence
Previous renal dysfunction	Renal dysfunction, renal failure, renal failure unspecified	Absence vs presence
Cerebral infarction	Cerebral infarction, cerebral stroke	Absence vs presence
Cancer	Cancer, tumors of the organs or blood systems	Absence vs presence
Cirrhosis	Liver cirrhosis, compensatory or decompensated cirrhosis	Absence vs presence
**Clinical information**		
NYHA classification	Assessed according to the NYHA Functional Classification<br>I: Cardiac disease, but no symptoms and no limitation in ordinary physical activity, e.g. no<br>shortness of breath when walking, climbing stairs etc.	II vs III vs IV

	II: Mild symptoms (mild shortness of breath and/or angina) and slight limitation during ordinary activity.<br>III: Marked limitation in activity due to symptoms, even during less-than-ordinary activity, e.g. walking short distances (20–100 m); comfortable only at rest.<br>IV: Severe limitations. Experiences symptoms even while at rest.	
Paroxysmal nocturnal dyspnea	Assessed at admission	Absence vs presence
Orthopnoea	Assessed at admission	Absence vs presence
Heart rate	Assessed at admission	In units of beats / minute
Systolic blood pressure	Assessed at admission	In units of mmHg
Diastolic blood pressure	Assessed at admission	In units of mmHg
Rales (>1/2 lung fields)	Assessed at admission	Absence vs presence
Jugular venous distension	Assessed at admission	Absence vs presence
Peripheral edema	Assessed at admission	Absence vs presence
**Imaging results**		
LVEF	The first value being within 2 days of onset admission by two-dimensional transthoracic echocardiography	In units of %
**Laboratory findings**		
B-type natriuretic peptide	The first value being within 2 days of onset admission	In units of pg/ml
Troponin I	The first value being within 2 days of onset admission	In units of ng/ml
Hemoglobin	The first value being within 2 days of onset admission	In units of g/L
C-reactive protein	The first value being within 2 days of onset admission	In units of mg/L
Alanine aminotransferase	The first value being within 2 days of onset admission	In units of IU/L

Blood urea nitrogen	The first value being within 2 days of onset admission	In units of mmol/L
Creatinine	The first value being within 2 days of onset admission	In units of μmol/L
Albumin	The first value being within 2 days of onset admission	In units of g/L
Serum sodium	The first value being within 2 days of onset admission	In units of mmol/L
Serum potassium	The first value being within 2 days of onset admission	In units of mmol/L
Uric acid	The first value being within 2 days of onset admission	In units of μmol/L
Glucose	The first value being within 2 days of onset admission	In units of mmol/L
**Treatment**		
Aldosterone antagonists	During a hospital stay	Used or not used
Loop diuretic	During a hospital stay	Used or not used
ACE-Is/ARBs	During a hospital stay	Used or not used
Beta-blockers	During a hospital stay	Used or not used
Anticoagulants	During a hospital stay	Used or not used
Aspirin	During a hospital stay	Used or not used
ADP-P2Y12 antagonists	During a hospital stay	Used or not used
Vasopressor	During a hospital stay	Used or not used

Variables were chosen based on a previous literature review and clinical evidence of risk factors for mortality, hospitalization for heart failure.
History of Medical History recorded using diagnosis codes (International Classification of Diseases, Ninth Revision, Clinical Modification.
Abbreviations:
NYHA, New York Heart Association
LVEF, left ventricular ejection fraction
ACE-Is/ARBs, angiotensin-converting enzyme inhibitors / Angiotensin Receptor Blockers
ADP, adenosine diphosphate

Table S2. Baseline characteristics of 871 patients: pre-imputation and post-imputation.

Variables	Pre-imputation	Post-imputation
Sex, Male	412 (47.30%)	412 (47.30%)
Age, years	75.30 (12.54)	75.30 (12.54)
BMI(kg/m^2)	24.34 (4.32)	24.34 (4.32)
Missing	31 (3.56%)	
Diabetes	370 (42.48%)	370 (42.48%)

Hypertension	605 (69.46%)	605 (69.46%)
Coronary artery disease	587 (67.39%)	587 (67.39%)
Previous heart failure	283 (32.49%)	283 (32.49%)
Atrial fibrillation	356 (40.87%)	356 (40.87%)
Previous renal dysfunction	158 (18.14%)	158 (18.14%)
Cerebral infarction	178 (20.44%)	178 (20.44%)
Cancer	96 (11.02%)	96 (11.02%)
Cirrhosis	11 (1.26%)	11 (1.26%)
NYHA classification		
II	242 (27.78%)	242 (27.78%)
III	401 (46.04%)	401 (46.04%)
IV	228 (26.18%)	228 (26.18%)
Paroxysmal nocturnal dyspnea	166 (19.06%)	166 (19.06%)
Orthopnoea	168 (19.29%)	168 (19.29%)
Heart rate (beats/min)	89.60 (22.60)	89.60 (22.60)
Systolic blood pressure (mmHg)	130.84 (24.60)	130.84 (24.60)
Diastolic blood pressure (mmHg)	72.65 (16.68)	72.65 (16.68)
Rales (>1/2 lung fields)	361 (41.45%)	361 (41.45%)
Jugular venous distension	168 (19.29%)	168 (19.29%)
Peripheral edema	559 (64.18%)	559 (64.18%)
LVEF (%)	51.51 (11.79)	51.51 (11.79)
B-type natriuretic peptide (pg/ml)	762.00 (323.50-1587.50)	786.00 (331.50-1601.00)
Missing	20(2.30%)	
Troponin I(ng/ml)	0.05 (0.04-0.10)	0.05 (0.04-0.10)
Missing	3(0.34%)	
Hemoglobin (g/L)	116.13 (24.62)	116.13 (24.62)
C-reactive protein (mg/L)	9.15 (3.90-25.28)	10.00 (4.00-27.14)
Missing	54(6.20%)	
Alanine aminotransferase (IU/L)	16.30 (11.20-27.58)	16.40 (11.20-27.85)
Missing	5(0.57%)	
Blood urea nitrogen (mmol/L)	8.10 (5.90-11.70)	8.10 (5.90-11.70)
Creatinine (μmol/L)	92.40 (72.90-127.45)	92.40 (72.90-127.45)
Albumin (g/L)	35.57 (4.87)	35.57 (4.87)
Missing	4(0.46%)	
Sodium (mmol/L)	138.43 (5.60)	138.43 (5.60)
Potassium (mmol/L)	4.23 (0.62)	4.23 (0.62)
Uric acid (μmol/L)	392.90 (297.00-514.20)	392.80 (296.65-514.20)
Missing	5(0.57%)	
Glucose (mmol/L)	7.18 (5.77-9.79)	7.17 (5.77-9.79)

Missing	5(0.57%)	
Aldosterone antagonists	643 (73.82%)	643 (73.82%)
Loop diuretic	804 (92.31%)	804 (92.31%)
ACE-Is/ARBs	332 (38.12%)	332 (38.12%)
Beta-blockers	602 (69.12%)	602 (69.12%)
Anticoagulants	259 (29.74%)	259 (29.74%)
Aspirin	376 (43.17%)	376 (43.17%)
ADP-P2Y12 antagonists	370 (42.48%)	370 (42.48%)
Vasopressor	86 (9.87%)	86 (9.87%)

Data are presented as frequencies (percentages) or mean (SD) or median (IQR)
Abbreviations:
IQR, interquartile range; SD, standard deviation
BMI, body mass index
NYHA, New York Heart Association
LVEF, left ventricular ejection fraction
ACE-Is/ARBs, angiotensin converting enzyme inhibitors / Angiotensin Receptor Blockers
ADP, adenosine diphosphate

Table S3 Comparison of the subgroup

subgroup	Male	Female	With coronary artery disease	Without coronary artery disease
C-Statistics	0.827	0.912	0.859	0.906
95%CI low	0.748	0.868	0.802	0.846
95%CI upp	0.906	0.955	0.916	0.967
Best threshold	-1.318	-3.066	-2.950	-3.511
Specificity	0.955	0.755	0.668	0.756
Sensitivity	0.559	0.911	0.907	0.929

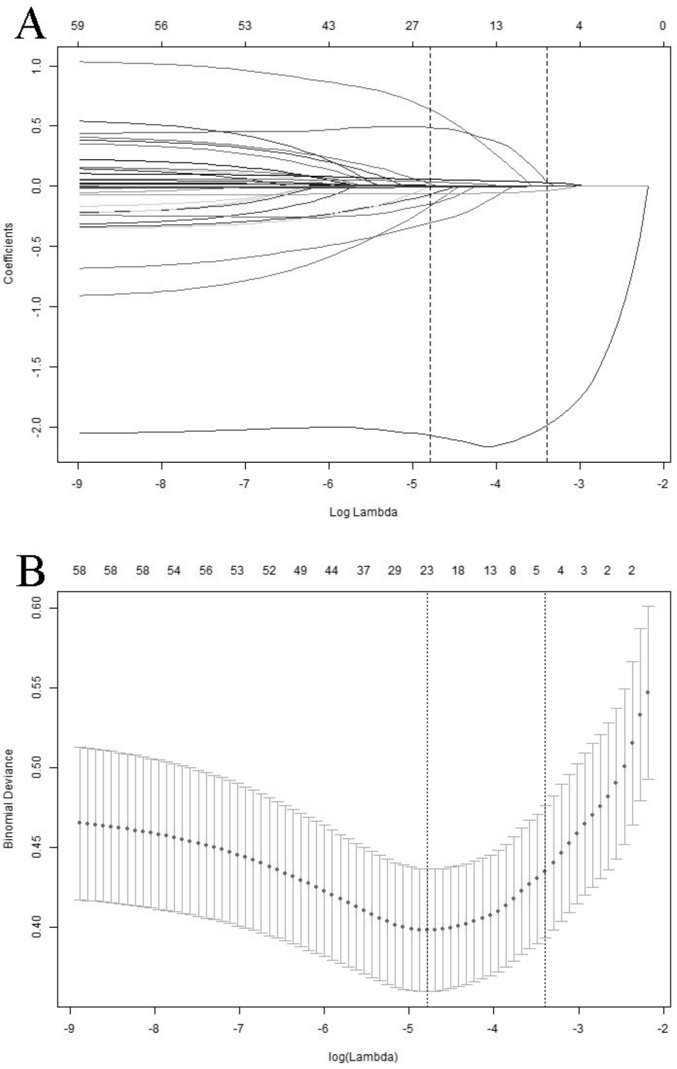

Figure S1 LASSO model profile plots. A, Tuning parameter (lambda: λ) selection in the LASSO model used 10-fold cross-validation via minimum criteria. B, LASSO coefficient profiles of the features against the log(λ).

Abbreviations:

LASSO, least absolute shrinkage and selection operator.
